# The mechanism of gut-lung axis in pulmonary fibrosis

**DOI:** 10.3389/fcimb.2024.1258246

**Published:** 2024-02-01

**Authors:** Yawei Dong, Lanlan He, Zhongbo Zhu, Fan Yang, Quan Ma, Yanmei Zhang, Xuhui Zhang, Xiping Liu

**Affiliations:** ^1^ Key Laboratory of Gansu Provincial Prescription Mining and Innovative Translational Laboratory, Gansu University of Chinese Medicine, Lanzhou, Gansu, China; ^2^ Gansu Provincial Traditional Chinese Medicine New Product Creation Engineering Laboratory, Gansu University of Chinese Medicine, Lanzhou, Gansu, China; ^3^ Respiratory Medicine, Affiliated Hospital of Gansu University of Chinese Medicine, Lanzhou, Gansu, China

**Keywords:** pulmonary fibrosis, gut-lung axis, microbiome, metabolite, immune regulation, pathogenic mechanism, related treatment

## Abstract

Pulmonary fibrosis (PF) is a terminal change of a lung disease that is marked by damage to alveolar epithelial cells, abnormal proliferative transformation of fibroblasts, excessive deposition of extracellular matrix (ECM), and concomitant inflammatory damage. Its characteristics include short median survival, high mortality rate, and limited treatment effectiveness. More in-depth studies on the mechanisms of PF are needed to provide better treatment options. The idea of the gut-lung axis has emerged as a result of comprehensive investigations into the microbiome, metabolome, and immune system. This theory is based on the material basis of microorganisms and their metabolites, while the gut-lung circulatory system and the shared mucosal immune system act as the connectors that facilitate the interplay between the gastrointestinal and respiratory systems. The emergence of a new view of the gut-lung axis is complementary and cross-cutting to the study of the mechanisms involved in PF and provides new ideas for its treatment. This article reviews the mechanisms involved in PF, the gut-lung axis theory, and the correlation between the two. Exploring the gut-lung axis mechanism and treatments related to PF from the perspectives of microorganisms, microbial metabolites, and the immune system. The study of the gut-lung axis and PF is still in its early stages. This review systematically summarizes the mechanisms of PF related to the gut-lung axis, providing ideas for subsequent research and treatment of related mechanisms.

## 1 Introduction

PF is not a singular ailment, rather, it is a condition characterized by diffuse alveolitis, disruptions in alveolar structure, and the extensive formation of scars. These factors hinder the efficient exchange of oxygen and carbon dioxide between the alveoli and pulmonary blood vessels, ultimately resulting in the development of interstitial lung fibrosis. Damage to alveolar epithelial cells, pathological fibroblast proliferation and transformation, and an excessive buildup of ECM are the hallmarks of this condition, which results in structural damage to lung tissue and a loss of function (Savin et al., 2022). Typically, PF occurs in the advanced stages of many interstitial lung diseases (ILD). ILD can be divided into four categories: idiopathic, autoimmune-related/connective tissue disease, exposure-related, and interstitial lung disease with cysts or airspace filling (Wijsenbeek et al., 2022). The common ones are idiopathic pulmonary fibrosis (IPF), systemic sclerosis (SSc), silicosis, radiological pulmonary fibrosis (RPF), drug-induced interstitial fibrosis, and cystic fibrosis (CF) (George et al., 2020). The pathogenesis of PF formation is complex, with a short median survival and high mortality rate after diagnosis, and there are still no effective advances in treatment. (Kinoshita and Goto, 2019). Hence, it is imperative to investigate and elucidate the underlying mechanisms of PF, as this is essential for expanding the array of therapeutic choices available to patients with this condition, enhancing their quality of life, and extending their lifespan.

The gut-lung axis is a bi-directional axis connecting the lungs and intestines based on the microbiota and metabolites that colonize the lungs and intestines, through the common mucosal immune system of the intestines and lungs, and through the blood and lymphatic systems between the intestines and lungs (Marsland et al., 2015; Shi et al., 2021). The composition and functional changes of the gut microbiota can affect the respiratory system through the common mucosal immune system, and microbial disorders in the respiratory tract can also affect the function of the digestive tract through immune regulation (Tamburini and Clemente, 2017). Through extensive and thorough examinations of the microbiome, there is a growing recognition of the role of gut microbiome metabolites in modulating the host (Cruz et al., 2021). Microbiota and their metabolites play an important role in various lung diseases, including PF, and in recent years have become a new frontier in the study of lung-related diseases (Zhang et al., 2020). In this article, we review the relevance of the gut-lung axis in PF diseases in the light of the latest advances in the mechanisms of PF at home and abroad, and systematically elucidate the current status of research on the mechanisms of the gut-lung axis in regulating PF diseases and the current status of treatment.

## 2 Related mechanisms of pulmonary fibrosis

### 2.1 Cellular-related mechanisms of pulmonary fibrosis

**Figure 1:**
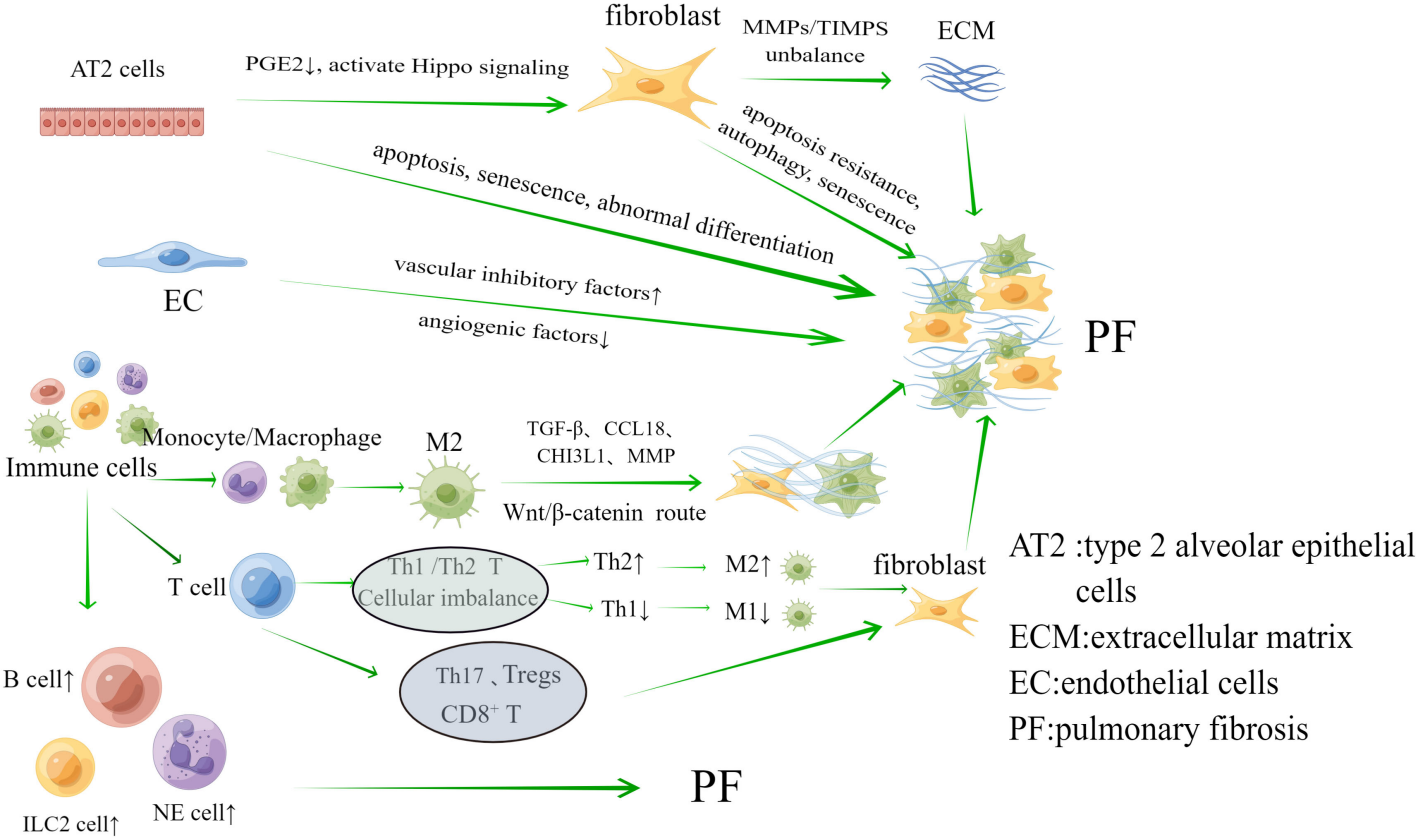
The main cells associated with the mechanism of pulmonary fibrosis are Alveolar epithelial cells, fibroblasts, endothelial cells and immune cells. They play an important role in the progression of pulmonary fibrosis.

The key pathomechanism of PF may be dysregulation of the interaction between inflammation and repair. Inflammation in PF may be accompanied by initial damage in the early stages of the disease, involving a series of complex interactions between soluble factors and cells (Heukels et al., 2019; Savin et al., 2022). Damage to tissues can also set off the body’s “repair response.” The key processes involved in the repair of this disorder are the ineffectiveness of alveolar re-epithelialization, activation of pulmonary fibroblasts and myofibroblasts, abnormal angiogenesis due to endothelial cell disorders, and unimpeded production of collagen and ECM (Moss et al., 2022; Savin et al., 2022). Hence, the involvement of alveolar epithelial cells, fibroblasts and myofibroblasts, endothelial cells (EC) and immune cells are crucial in the development of PF, as shown in **Figure 1**.

#### 2.1.1 Alveolar epithelial cells

Alveolar epithelial cells are divided into type 1 and type 2, among which type 1 alveolar epithelial (AT1) cells primarily function to maintain alveolar structure. As the primary progenitor cells of damaged AT1 cells, type 2 alveolar epithelial (AT2) cells have the potential for unlimited proliferation, with AT2 cells playing a major role in PF (Parimon et al., 2020). (I) Apoptosis, senescence, abnormal differentiation and impaired renewal capacity of AT2 cells are among the key factors in the development of PF. Aging, endoplasmic reticulum stress, mitochondrial dysfunction, and telomere shortening can lead to the inability of AT2 cells to effectively repair damaged epithelia. During this process, there are problems with a number of cellular processes, including matrix metalloproteinase 7 (MMP7), integrins αVβ6, cellular senescence, and epithelial-mesenchymal transition (EMT) (Moss et al., 2022). Typical examples are cell aging and EMT. The aging process includes activation of ataxia telangiectasia mutated/nuclear factor κB (NF-κB), ATM/NEMO signaling, p53 and phosphatidylinositol 3-kinase (PI3K) -Akt signaling (Nicolae et al., 2018; Tsoyi et al., 2021; Yao et al., 2021). The main thing that starts EMT is transforming growth factors-β (TGF-β), which change alveolar epithelial cells into myofibroblasts. EMT may be linked to different signaling pathways, including Smads, Wnt, Notch, nuclear factor-kB, and Sonic hedgehog (Shh) (Salton et al., 2019). (II) Abnormal regulation of fibroblasts caused by apoptosis and damage to alveolar epithelial cells is also an important aspect of PF development. On the one hand, alveolar epithelial cells undergo excessive apoptosis, leading to a decrease in the negative feedback factor prostaglandin E2 (PGE2) level, which is anti-fibrosis locally, and fibroblasts lose inhibition, causing an excessive inflammatory reactions and the initiation of abnormal repair function (Epa et al., 2015). On the other hand, damaged AT2 cells stimulate fibroblast proliferation by activating Hippo signaling to produce target genes (Gokey et al., 2018), and also release mtDNA to aid fibroblast activation in IPF (Bueno et al., 2019).

#### 2.1.2 Pulmonary fibroblasts and myofibroblasts

Fibroblasts and myofibroblasts are the main effector cells of PF. Fibroblasts overproliferates at the site of injury and differentiates into myofibroblasts during abnormal wound healing and connective tissue repair. Both of these cells can release large amounts of ECM, which further leads to PF. Under the stimulation of growth factors and cytokines, such as connective tissue growth factor (CTGF), platelet-derived growth factor (PDGF), TGF-β1, IL-1β, IL-6, IL-13, and IL-33 (Kendall and Feghali-Bostwick, 2014), as well as aberrant activation of pro-fibrotic pathways including Wnt (Königshoff et al., 2008; Hamburg-Shields et al., 2015), TGF-β, Shh (Cigna et al., 2012), and Notch (Liu et al., 2009; Chanda et al., 2019), fibroblasts undergoes apoptosis resistance, autophagy, and senescence that in turn trigger PF (Moss et al., 2022). At the same time, when the balance of matrix metalloproteinases (MMPs) and tissue inhibitors of matrix metalloproteinases (TIMPS) is upset, it leads to abnormal aggregation in the ECM. This is another important way that fibroblasts is involved in the PF process (Mahalanobish et al., 2020).

#### 2.1.3 Endothelial cells

Angiogenesis is essential for wound repair, and EC are involved in this process. Observations in fibrotic lungs have shown a reduced vascular distribution within fibrotic lesions. Vascular inhibitory factors that are highly expressed and angiogenic factors that are lowly expressed are two important ways that fibroblast foci have less blood flow. One important factor in angiogenesis is vascular endothelial growth factor (VEGF). Research has found a decrease in VEGF protein levels in IPF lung biopsy (Murray et al., 2017). Vasculosuppressive factors include pigment epithelium-derived factor (PEDF), which effectively inhibits TGF-β1-stimulated fibroblast activation and also inhibits the TGF-β1/smad pathway by up-regulating PPAR-γ activity, which promotes lung fibrosis (Qin et al., 2022). It is thought that when there is an imbalance in angiogenesis, microvascular remodeling happens. EC go through EndMT through TGF-β and Ras/mitogen-activated protein kinase signaling, changing into fibroblasts and causing PF (Hashimoto et al., 2010).

#### 2.1.4 Immune cells

In the complex regulation between inflammation and repair, there is a significant aggregation of immune cells (Wynn and Vannella, 2016). Innate immune cells include monocytes, macrophages, neutrophils, and innate lymphoid cells. (I) Macrophages and monocytes: In reaction to inflammation and damage, the resident alveolar macrophage (AM) was activated, while a recruited monocyte-derived group aids in alveolar recovery. Monocyte counts and monocyte chemokine CCL2 were elevated in IPF, a process that caused poor prognosis (Kreuter et al., 2021). Monocyte-derived AM expresses pro-fibrotic genes and localizes to fibrotic areas adjacent to fibroblasts and damaged epithelial cells (Joshi et al., 2020). The essential function of AM lies in preserving lung equilibrium through the clearance of apoptotic cells and debris, the control of wound healing, and their contribution to instigating immune responses against pulmonary pathogens (Hussell and Bell, 2014). The major role in lung fibrosis is played by M2-type macrophages, which can promote fibrosis through a variety of mechanisms, including the production of TGF-β, CCL18, CHI3L1, MMP, and activation of the Wnt/β-catenin pathway leading to fibroblast activation, myofibroblast differentiation, and ECM remodeling (Shenderov et al., 2021). (II) Neutrophils: when neutrophils clump together, they release pro-inflammatory cytokines and reactive oxygen species that may make tissue remodeling worse in lung injury (Mayadas et al., 2014). Researchers have found that people with IPF have higher levels of neutrophils and eosinophils in their bronchoalveolar lavage fluid (BALF). This higher level is linked to the concentration of the chemokine CCL18 (Schupp et al., 2015). (III) ILCs: Type 2 innate lymphoid cells (ILC2) are the main type of ILCs in the lungs. The ILC2 subtype releases common pro-fibrotic mediators, such as IL-4, IL-5, IL-9, and IL-13, which play a role in the development of PF (Mikami et al., 2018). One study found that IL-13 released by ILC2 was sufficient to drive collagen deposition in the lungs of attacked mice (Hams et al., 2014). Pro-fibrotic cytokines released by AM and EC induce ILC2 cell activation, enhance IL-13 and TGF-β production, and promote lung fibrosis (Li et al., 2014).

Adaptive immune cells include T cells and B cells. (I) T lymphocytes: firstly, Th1/Th2 CD4^+^ T cell imbalance is one of the pathogenic mechanisms of PF. IL-4 and IL-13 producing Th2 cells promote fibroblast differentiation and M2 macrophage production, whereas IL-12 and IFN-γ producing Th1 cells inhibit fibroblast differentiation and promote M1 macrophage production (Shao et al., 2008; Gordon and Martinez, 2010). Secondly, Th17 CD4^+^T cells can promote fiber formation (Lei et al., 2016). In SSc patients, the frequency of Th17 cells increases and serum IL-17 levels increase (Rodríguez-Reyna et al., 2012). This is supported by the fact that IL-17A and IL-1β levels are higher in the BALF of people with IPF (Wilson et al., 2010). Thirdly, early reports suggested a decrease in the number of Tregs in the blood and BALF of patients with IPF (Kotsianidis et al., 2009). However, subsequent studies have contradicted this finding and instead reported an increase in the number of Tregs in the blood and BALF of IPF patients, and the increase in the number of Tregs correlated with an increase in Th17 cells, a decrease in Th1 cells differentiation, and an increase in TGF-β (Galati et al., 2014; Hou et al., 2017; Chakraborty et al., 2018). Fourthly, CD8+ T cells are spread out widely in the lung parenchyma and alveolar walls. People with IPF who have more CD8+ T cells in lung biopsies tend to have lower total lung capacity and forced vital capacity. (II) B lymphocytes: B lymphocytes have a detrimental impact on the advancement of IPF, and there is an upregulation of genes associated with B-cell markers and distinct chemokines (including CXCL13, CXCR5, CCR6, and CCR7) in the lung tissues of individuals suffering from IPF (DePianto et al., 2015; Havenar-Daughton et al., 2016). In patients with IPF, B-cell activating factor acts by regulating B-cell survival, and its concentration in the plasma of IPF patients correlates with disease progression (Xue et al., 2013). B-cell activating factor has been reported to be a key factor in bleomycin- and IL-7-mediated experimental PF (François et al., 2015). Other studies have shown that B cells are involved in IPF pathogenesis by targeting B cell receptor (BCR) signaling (Neys et al., 2021).

### 2.2 The gut-lung axis can supplement the relevant mechanisms of pulmonary fibrosis

Many studies have been done on the causes of PF, including how different cytokines are activated, how immune cells are involved, and how different fibroblast subtypes change, leading to lung epithelial cells that don’t work as well, EC disorder, and activated fibroblasts that make collagen and ECM. However, many of the mechanisms involved are still not fully understood. The emergence of a new perspective on the gut-lung axis provides a supplement and intersection for the study of cellular mechanisms related to PF and provides new ideas for the study and treatment of the pathogenesis of PF.

**Figure 2:**
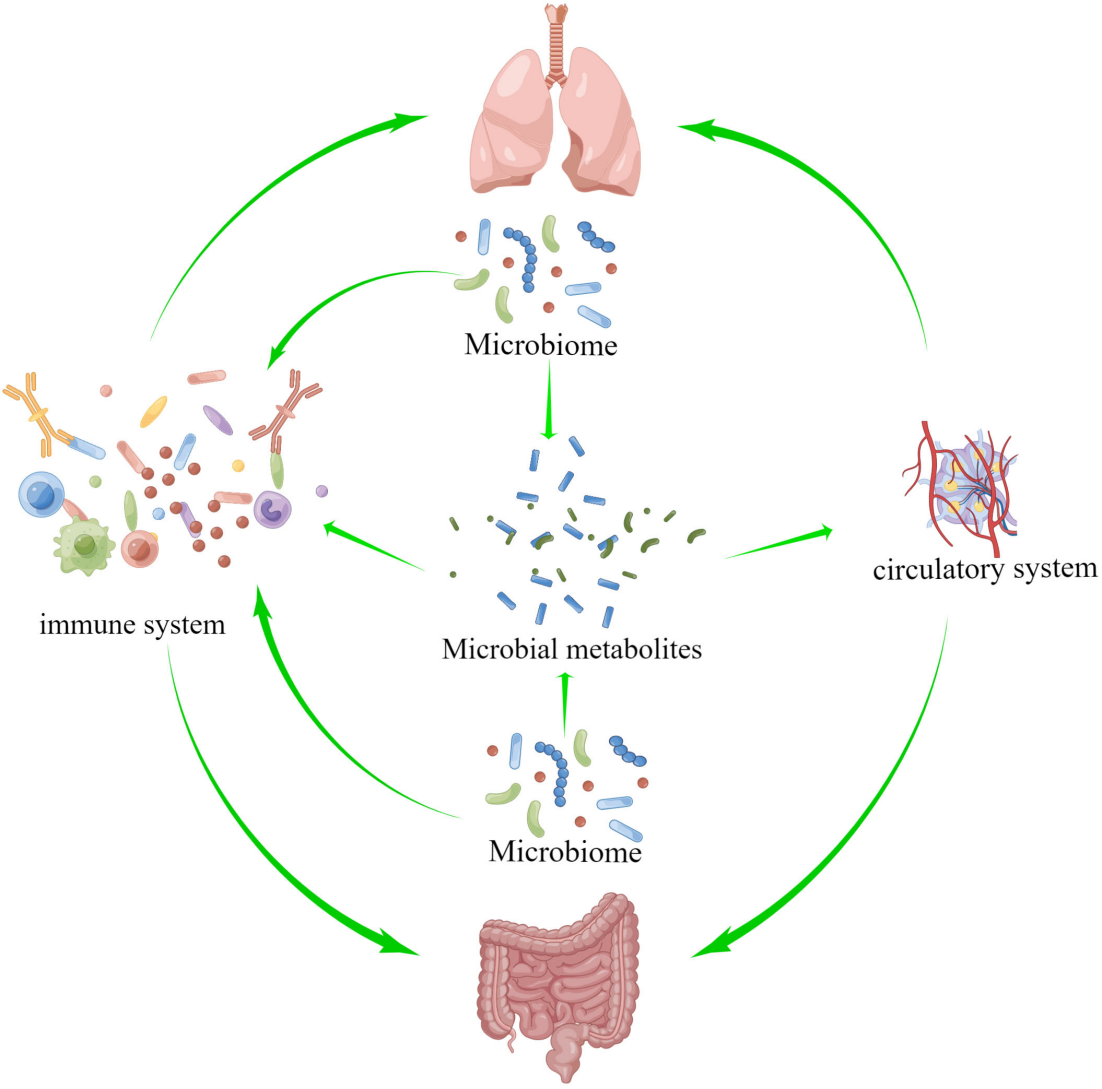
The theory of the gut-lung axis is based on the material basis of microorganisms and their metabolites, and on the gut-lung circulatory system and the common mucosal immune system as a bridge to exert gut-lung interactions.

The theory of the gut-lung axis is based on the material basis of microorganisms and their metabolites, the gut-lung circulatory system and the common mucosal immune system as a bridge to exert gut-lung interactions, as shown in **Figure 2**. Microbial communities colonizing the mucous membranes of the digestive and respiratory systems are an important biological material basis for exerting regulatory effects and strengthening the lung-gut liaison (Chunxi et al., 2020; Tang et al., 2021). The blood and lymphatic system between the lung and the intestine is connected, which is part of the bridge of the gut-lung axis, and the metabolites of microorganisms can enter the circulation and reach the lungs, and affect the progression of diseases in a variety of ways, such as inflammation and cell signal transduction (Marsland et al., 2015; Dickson et al., 2018). Short-chain fatty acids (SCFAs) are made when the microbiota in the intestines break down fiber, and once they reach the intestine lumen, they trigger an immune response and provide energy to the colonocytes, especially butyric acid. If the gastrointestinal tract doesn’t use all of the SCFAs, they go through the portal vein and are sent to the liver to be broken down. SCFAs that are not metabolized in the liver enter the peripheral circulation and bone marrow. There, they affect the growth of immune cells and the metabolism and function of organs and tissues outside of the intestines, like the lungs (Boets et al., 2017; Wypych et al., 2019). Through the mucosal immune system, the mucosal architecture in the respiratory and gastrointestinal tracts provides a safe haven for microbial communities to survive and protects the organism from pathogens (Verschuere et al., 2011; Allais et al., 2016). Growing evidence suggests that microorganisms and their metabolites are involved in mucosal immune development and are known to be important innate immune system modulators with a key role in maintaining immune system homeostasis (Budden et al., 2017). There are several pathways of gut-lung axis immune communication: (I) Certain elements within the gut microbiota, such as lipopolysaccharides (LPS), have the capacity to directly augment the host’s immune response. (II) The gut microbiota exerts indirect effects through: (i) the introduction of microbial metabolites like SCFAs and amino acids into the bloodstream, subsequently influencing immune cells. (ii) The activation of immune cells originating from the bone marrow, provoking immune responses in the lungs. (iii) The migration of intestinal immune cells from the gut to the lungs through the circulation, thereby influencing pulmonary immunity (Chioma et al., 2022; Ma et al., 2022). The intestine and lungs are based on microorganisms and metabolites, and interact and constrain each other through the circulatory and immune systems of the intestine and lungs, thereby achieving bidirectional regulation. In the study of mechanisms relating to lung diseases, the gut-lung axis is crucial, and it also regulates PF.

Pulmonary microbiota and metabolites cause PF through various fibrogenic factors, activation of fibrogenic pathways and activation of various immune cells, such as macrophages, neutrophils and other immune cells, through the circulatory system and the immune system to induce apoptosis of lung epithelial cells, EMT, activation of lung fibroblasts and other mechanisms. In addition to the local activation of immunity in the gut, intestinal microorganisms and metabolites can also activate the immune system through the peripheral circulation and secrete cytokines, etc., to cause lung fibrosis. The immune and circulatory systems serve as a bridge between these processes. The following will explore the mechanisms related to the gut-lung axis and PF in detail.

## 3 Gut-lung axis and pulmonary fibrosis

PF is regulated by a mechanism related to the gut-lung axis. This section introduces three aspects: microorganisms, metabolites and immune response. Since immune response is an intermediate process of PF caused by microorganisms and metabolites, it will be introduced into the mechanisms related to microorganisms and metabolites.

### 3.1 Microbiome and pulmonary fibrosis

#### 3.1.1 Pulmonary microorganisms and pulmonary fibrosis

For a long time, human lungs have been considered a sterile environment, but research has shown that the biomass of lung microbiota is low and mainly composed of oral symbiotes. Inhalation of oropharyngeal or gastroesophageal contents is the primary means by which bacteria reach the lower respiratory tract and plays an important role in lower respiratory mucosal immunity. The six principal genera of bacteria that have been identified in the lungs are *Prevotella*, *Streptococcus*, *Veillonella*, *Fusobacterium*, *Porphyromonas*, and *Neisseria* (Wypych et al., 2019). Maintenance of these lower biomasses is achieved through rapid clearance by a variety of physiologic mechanisms, with coughing, ciliary transport, and the innate immune system being the three primary lung clearance mechanisms (Natalini et al., 2023). The microbiome in the lungs changes all the time, with brief periods of exposure to oral commensals followed by fast clearance. This exposure to microorganisms and fast clearance can reach a state of dynamic equilibrium. When this dynamic balance is dysregulated, it activates the immune system, which in turn has a major impact on the development of respiratory diseases.

IPF is a typical type of PF that is also affected by microorganisms. A large number of patients with IPF have changes in the microorganisms in the BALF. It was found that the BALF of IPF patients had a decrease in microbial diversity, increased in the Firmicutes and Bacteroidetes, while the phylum Proteobacteria decreased, which was correlated with IPF indicators (Takahashi et al., 2018). Compared with healthy individuals, IPF patients show increased bacterial load and decreased diversity, and the BALF of IPF patients increased at the species level, including *Hemophilus*, *Streptococcus*, *Neisseria* and *Veillonella* spp. (Molyneaux et al., 2014; O'Dwyer et al., 2019). Another trial showed that lung flora exacerbates disease progression during acute exacerbations of IPF. At the time of diagnosis, the increased relative abundance of *streptococcus* and *staphylococcus* genera may indicate rapid progression of the disease (Han et al., 2014). BALF from IPF patients stimulated the growth of *Staphylococcus aureus* (*S aureus*) in an *in vitro* assay compared to normal controls, and levels of interleukin 1 receptor antagonist (IL1RA) were statistically significantly higher in BALF (Harrison, 2009). It has been shown that the lung microbiota plays a role in regulating Th1/Th2 during fibrotic injury, with an enhanced response of FOXP3 ^+^ T-regulatory cells, which can promote collagen deposition (O'Dwyer et al., 2019), and FOXP3 ^+^ T-regulatory cells can facilitate lung fibrosis by stimulating fibroblasts through the secretion of PDGF-B (Lo Re et al., 2011). Huang and others explored host immune responses and microbial interactions using BALF from IPF patients and found that they correlated with fibroblast function and leukocyte phenotype (Huang et al., 2017). Increased *Streptococcus* abundance was associated with increased NOD-like receptor immune response signaling, expression of the Toll-like receptor (TLR) immune pathway was associated with fibroblasts, lymphocytes expressing C-X-C chemokine receptor 3 CD8 were significantly associated with *staphylococcus*. The down-regulation of several innate immunity receptor genes in peripheral blood mononuclear cells (PBMCs) was correlated with lung microbial diversity in patients with IPF. These results validate the relationship between host immunity and lung microbiota (**Table 1**).

**Table 1:** Pulmonary microorganisms and pulmonary fibrosis.

Detection object	comparator	Extract target	Experimental methods	result	refer
IPF patients and conventional pulmonary fibrosis mouse models	A sterile mouse model of pulmonary fibrosis	BALF	16S rRNA gene sequencing	Increased bacterial load and decreased diversity	(Molyneaux et al., 2014)
IPF patients	Healthy smokers, non-smokers, and those with moderate chronic obstructive pulmonary disease	BALF	16S rRNA gene sequencing	Increased bacterial load, more abundant Haemophilus, Streptococcus, Neisseria and Veronicus	(O'Dwyer et al., 2019)
IPF patients and mice with bleomycin induced pulmonary fibrosis	Physiological saline group	BALF	16S rRNA gene sequencing	increases in the Firmicutes and Bacteroidetes (Streptococcaceae, Veillonellaceae, and Prevotellaceae families) and a decrease in the phylum Proteobacteria were involved in the reduction of diversity. And the loss of diversity is related to IPF index.	(Takahashi et al., 2018).
IPF patients	CHP subjects, IPF subjects, and control subjects	BAL	Bacterial DNA is isolated and quantified through quantitative PCR,16S rRNA gene sequencing	At the phylum level, Firmicutes dominates in IPF, while Proteobacteria is significantly lower than CHP; At the genus level, the burden of Staphylococcus in CHP increases, while the burden of Actinomyces and Veillonella in IPF increases.	
IPF patients	–	BALF	Isolation of DNA, 454 Pyrosequencing	The increase in relative abundance of Streptococcus and Staphylococcus may be related to rapid disease progression	(Han et al., 2014)
IPF patients	Wegener’s granulomatosis (WG) patients and normal controls	BALF	Cultivating Staphylococcus aureus using BALF from IPF patients	BALF stimulation of Staphylococcus aureus growth and elevation of interleukin-1 receptor antagonist (IL1ra) in IPF patients	(Harrison, 2009)
IPF patients	–	BALF、Lung fibroblasts obtained through bronchial biopsy	Isolation of peripheral blood mononuclear cell and RNA extraction16S rRNA gene sequencing、Lung fibroblast culture through bronchial biopsy	The increase in microorganisms correlates with fibroblast function and leukocyte phenotype	(Huang et al., 2017)

From the above studies, it is summarized that in the lungs of patients with IPF, there is a decrease in microbial diversity and an increase in bacterial load. At the phylum level, there was an increase in Firmicutes, Bacteroidetes and a decrease in Proteobacteria. At the genus level, *Haemophilus*, *Streptococcus*, *Neisseria*, *Veillonella* spp., *Staphylococcus* and *Streptococcus* genera were increased. In terms of immune response, microorganisms in the lungs of IPF patients can modulate Th1/Th2 and enhance FOXP3^+^ T regulatory cells. Staphylococci are significantly associated with lymphocytes expressing CXCR3^+^ CD8^+^ T cells. Microorganisms modulate the immune system to cause PF with the involvement of multiple immune pathways that promote collagen deposition and fibroblast activation.

#### 3.1.2 Intestinal microbes and pulmonary fibrosis

The intestinal tract contains a wide variety of microorganisms, including bacteria, fungi, phages, and viruses. In a healthy condition, there is a harmonious equilibrium between the gut microbiota and the host organism, which supports the normal functioning of physiological processes. The microbiome plays a crucial role in shaping the development of the host’s innate and adaptive immune systems, while the immune system itself manages the mutually beneficial relationship between the host and microorganisms. The dynamic disturbance of the gut microbiota has the potential to disrupt immunity, circulation, and metabolism, and this can result in the pathophysiology of lung disorders. Numerous studies have shown changes in intestinal flora in patients and mice with PF that correlate with indicators of PF. The researchers analyzed a database of 189 IPF patients living in the Modena area, consisting of 148 males (78.3%) and 41 females (21.7%). From this database, they were able to identify 44 patients (36 males, 81.8%; 8 females, 18.2%) who had a confirmed diagnosis of a gastrointestinal disease based on their histological features (Stefania et al., 2023). Experiments conducted on mice have shown that early-life exposure to antibiotics, leading to disruption of the gut microbiome, can promote the development of skin and lung fibrosis later in life (Ho and Varga, 2017). SSc is an autoimmune connective tissue disease characterized by immune dysregulation and progressive fibrosis, and intestinal microecological dysbiosis is prevalent in patients with SSc, with cross-sectional analyses revealing that 75.5% of SSc patients exhibited abnormal changes in intestinal flora, which were highly correlated with gastrointestinal inflammatory responses, and that the degree of dysbiosis was particularly severe in patients with PF (Andréasson et al., 2016). Topoisomerase I peptide-loaded dendritic cell immunization induces SSc-like disease, with changes in the gut microbiota and progressive skin and lung fibrosis. Oral administration of streptomycin early in life revealed an elevated ratio of *Bacteroidetes*/*Firmicutes* in the intestines of SSc mice, dysregulation of lung T-cell responses and exacerbation of PF (Mehta et al., 2017).

CF is the result of mutations in the *CFTR* gene and follows an autosomal recessive inheritance pattern. Several cross-sectional studies have shown that the gut microbiota of patients with CF is closely related to their lung function, disease progression, and severity (Burke et al., 2017; Coffey et al., 2019). In addition, patients with CF demonstrate a decrease in gut microbial diversity (Françoise and Héry-Arnaud, 2020), as well as a decrease in the abundance of bacteria that produce butyrate, such as Ruminococcaceae and *Butyricimonas*, and an increase in the abundance of other taxa, such as Actinobacteria and *Clostridium* (Dayama et al., 2020).

Silicosis is an occupational disease caused by prolonged inhalation of silica, and its severe pathological change is fibrosis of the lungs. By studying the intestinal microflora of silicosis patients, it was found that the abundance of Firmicutes and Actinobacteria decreased significantly, as well as lower levels of *Devosia*, *Clostridiales*, *Alloprevotella* and *Rikenellaceae_RC9*. *Lachnospiraceae* and *Lachnoclostridium* levels were increased (Zhou et al., 2019).

Researchers induced two mouse models of PF using bleomycin (BLM) and silica dust, and collected the feces of these mice for 16S rRNA gene sequencing technology and non-targeted metabolomics analysis of their gut microbiome. The results showed that PF mice had significant differences in 412 genera of gut microbiota and 26 metabolites compared to the control group. Seven gut microbiota and nine metabolites stood out as being very typical. The 16 biological and metabolic markers had a strong connection with well-known signs of fibrosis, such as levels of hydroxyproline, type I collagen, and fibronectin (Gong et al., 2021). It was found that the microecological balance of intestinal flora was disrupted in BLM-induced PF mice, such as *Catenibacterium* and *Lactobacillus*, which decreased significantly, while the relative abundance of *Verrucomicrobiales* and *Enterobacteriales* increased significantly. In terms of metabolic function prediction, BLM significantly increased the degradation of valine, leucine and isoleucine as well as the metabolism of glycine, serine and threonine in the gut microbiota, compared with the normal and sham groups (Quan et al., 2022). These alterations could be associated with the apoptosis of lung tissue cells, including alveolar epithelial cells, as well as the disruption of alveolar structure and excessive production of ECM, all stemming from the activation of the TGF-β/SMAD and caspase-3 pathways.

Zhan and others found that changes in the intestinal microbiota and their metabolites can activate various immune and non-immune cells in the host, induce inflammatory responses, and stimulate the production of a large amount of ECM components by mesenchymal cells, which is a common pathogenesis leading to fibrotic lesions in other distant organs outside the intestine (Zhan et al., 2021). The NF-κB signaling pathway is vital for preserving immune balance in the intestine, as it enhances mucosal immunity and safeguards against the development of chronic inflammation (Zaph et al., 2007). The activity of this pathway is regulated by the degradation of NF-κB proteins induced by TLR on the cell surface and by cytokine or pathogen-induced phosphorylation (Oeckinghaus and Ghosh, 2009). Both streptozotocin-induced diabetic mice and antibiotic-induced dysbiosis mice exhibit gut and lung microbiota dysbiosis, thickening of alveolar walls, and fibrosis, along with the activation of the NF-κB signaling pathway in the lungs. However, these conditions are significantly improved by fecal microbiota transplantation, and the use of Berberine and Baicalin can also correct gut and lung microbiota dysbiosis in streptozotocin-induced diabetic mice (Wang et al., 2021a) (**Table 2**).

**Table 2:** Intestinal microbes and pulmonary fibrosis.

Types of pulmonary fibrosis	Detection object	comparator	Changes in gut microbiota	result	conclusion	refer
Pulmonary fibers associated with scleroderma (SSc)	A Scleroderma Mouse Model with Early Administration of Antibiotics	Scleroderma mouse model without antibiotic administration	–	Increased skin and lung fibrosis in later years	Changes in gut microbiota caused by early antibiotic exposure in life may be the pathogenesis of scleroderma	(Ho and Varga, 2017)
	SSc mice who took streptomycin orally in early life	SSc mice without oral streptomycin	Ratio of Bacteroides/Firmicutes↑	Aggravation of pulmonary fibrosis and dysfunction of lung T cell response	The changes of intestinal microbiome caused by streptomycin will aggravate the fibrosis of SSc lung region	(Mehta et al., 2017)
	SSc patients	Healthy control population	(75.5%) patients exhibit ecological imbalance of gut microbiota	The degree of microbial imbalance is particularly severe in patients with pulmonary fibrosis and is highly correlated with gastrointestinal inflammatory response	Severe imbalance of gut microbiota in SSc patients with pulmonary fibrosis and high correlation with gastrointestinal inflammatory response	(Andréasson et al., 2016)
Cystic fibrosis (CF)	Children with cystic fibrosis (CF)	healthy control	genera Escherichia ↑, Shigella ↑, Enterobacter ↑, Morganella ↑, Veillonella ↑, Fusobacterium ↑, Ruminococcaceae↓,Alistipes ↓	Correlation between gut microbiota and growth index and lung function	Future gastrointestinal treatment for CF should explore microbial changes	(Burke et al., 2017)
	CF patients with cystic fibrosis	healthy control	Microbial diversity reduced, Firmicutes ↑, Bacteroidetes ↓	Reduced microbial diversity is associated with pulmonary dysfunction	Compared to healthy controls, the microbial diversity of CF patients decreases	(Coffey et al., 2019)
	CF patients with cystic fibrosis	healthy control	Intestinal microbial diversity ↓, Ruminococcaceae ↓, Butyricimonas ↓, Actinobacteria ↑, Clostridium ↑	–	Biomarkers may serve as targets for patients with cystic fibrosis	(Dayama et al., 2020)
A mouse model of pulmonary fibrosis	A mouse model of pulmonary fibrosis	Normal group and sham operation group	412 genera of gut bacteria and 26 metabolites showed significant differences compared to the control group	7 gut microbiota and 9 metabolites are representative and highly correlated with classic fibrosis indicators	Pulmonary fibrosis is highly correlated with gut microbiota and metabolites in a mouse model	(Gong et al., 2021)
	Bleomycin induced mouse PF model	Normal group and sham operation group	The microecological balance of intestinal flora is destroyed. Catenibacterium ↓, Lactobacillus↓, Verrucomicrobiales ↑, Enterobacteriales ↑	Apoptosis of alveolar epithelial cells and other lung histiocyte, destruction of alveolar structure and ECM deposition may be associated with TGF- β/ SMAD and caspase-3 pathway related	The gut microbiota changes related to PF and the concept of "gut lung axis" in BLM induced mouse models may provide an alternative treatment strategy for PF	(Quan et al., 2022)
Patients with PF caused by silica	Patients with PF caused by silica	Healthy subjects	proteobacteria↑, actinbacteria↑, verucomicrobia↑, Megamonas ↑, Lachnospiraceae ↑, Lachnoclostridium ↑, Parabacteroides ↑; Firmicutes ↓, Actinobacteria ↓, Bacteroides ↓, Escherichia-Shigella↓	–	Changes in gut microbiota in patients with silicosis	(Zhou et al., 2019)
Mouse model with fibrotic changes in the lungs	Streptozotocin-induced diabetic mice and antibiotic-induced intestinal dysbiosis mice with altered lung fibrosis	Saline group Fecal microbial transplantation treatment group Baicalin treatment group	Dysbiosis of the gut and lung microbiota	NF-κB mucosal immune, inflammatory signaling pathways were activated in the lungs of mice with pulmonary fibrosis, with alveolar wall thickening and fibrotic changes, and these processes were ameliorated by fecal microbial engraftment and baicalin.	Dysbiosis of gut and lung microbiota can activate NF-κB inflammatory fluxes causing pulmonary fibrosis, which can be ameliorated by fecal microbial transplantation and baicalin.	(Wang et al., 2021a)
A mouse model of pulmonary fibrosis	Pulmonary fibrosis was induced by bleomycin in two environments: ABSL-1 and ABSL-2	Induction of pulmonary fibrosis with bleomycin in GF (no microbial) environment	Metagenomic analysis reveals no notable distinctions between ABSL-1 and ABSL-2 lung microbiota, whereas greater microbial diversity, with increased Bifidobacterium and Lactobacilli, is present in ABSL-1 compared to ABSL-2 gut microbiota	ABSL-2 mice have severe pulmonary fibrosis, ABSL-1 mice have mild pulmonary fibrosis, and GF mice do not have pulmonary fibrosis. There are no microorganisms in the lungs of mice in all three environments.	The induction of pulmonary fibrosis in mice by bleomycin requires the presence of microorganisms, and the severity of pulmonary fibrosis is related to the diversity of gut microbiota and not related to lung microbiota.	(Chioma et al., 2022)

In PF-related diseases, we summarize the changes in intestinal flora in patients with SSc and CF, and in a mouse model of PF induced with silica and BLM. Clinical trials have found changes in the intestinal flora in patients with SSc, and animal experiments have found elevated proportions of *Bacteroidetes* and *Firmicutes* and dysregulated T-cell responses in the intestines of SSc mice. In individuals with CF, a reduction in gut microbial diversity, a decline in the presence of beneficial butyrate-producing bacteria, and an increase in populations like Actinobacteria and Clostridium have been observed. Reduced Firmicutes and Actinobacteria have been found in patients with silicosis. Animal experiments with BLM and silica dust-induced PF mice have shown alterations in gut microbes and metabolites, with the changes possibly related to apoptosis, destruction of alveolar structure, and overproduction of ECM. In terms of immune response, the dysregulation of gut flora and its metabolites can activate immune cells and immune signaling pathways, inducing an inflammatory response and causing PF.

#### 3.1.3 The effect of gut microbiota on lung microbiota

To further explore the mechanisms involving the impact of gut microorganisms on lung microorganisms and the development of PF, Chioma and colleagues exposed mice to BLM in three distinct environments: GF (germ-free, devoid of microorganisms), ABSL-1 (Animal Biosafety Level 1, where experiments exclude infectious agents and mice are only exposed to commensal organisms), and ABSL-2 (Animal Biosafety Level 2, where experiments involve the presence of an infectious agent in the environment, posing a moderate potential risk to personnel). GF mice were found to have no microorganisms in their intestines and no PF. ABSL-2 mice had the heaviest fibrosis and lower gut microbial diversity. ABSL-1 mice had less severe fibrosis and higher microbial diversity was present. Higher microbial diversity and increased *Bifidobacteria* and *Lactobacillus* were found in ABSL-1 compared to the ABSL-2 gut microbiota. Further transplantation of the ABSL-2 feces into GF mice showed heavier fibrotic lung disease, but transplantation of the ABSL-1 feces into GF mice did not cause significant PF. It is noteworthy that no significant differences in lung microbial diversity were detected in the BLM-treated mouse model. This is different from previous reports on the subject and may be related to the excessive number of influencing factors in the natural environment (Chioma et al., 2022).

When mice were modelled in different microbial environments, PF was heaviest in environments with infectious agents and correlated with gut microbial diversity. But the degree of lung fibrosis and intestinal microbial diversity did not correlate well with lung microbial diversity. This experimental approach controls and excludes the influence of other possible factors, and such an approach is particularly good, but there are still relatively few relevant studies, and it is an important direction for a better study of the gut-lung axis in the future.

### 3.2 Metabolites and pulmonary fibrosis

#### 3.2.1 The regulation of pulmonary microbial components and secretions related to the mechanism of pulmonary fibrosis

A small number of microorganisms are known to be present in the lungs, and when the balance between the microorganisms in the lungs and the body’s clearance mechanisms is disrupted, the microbial components and secreted substances respond to the body and cause PF. LPS, which is a constituent of the membrane of Gram-negative bacteria, can be recognized by the membrane receptor TLR4 and induces activation of fibroblasts and deposition of collagen (He et al., 2009; de Souza Xavier Costa et al., 2017). Additionally, experimental studies have found that LPS can promote proliferation of lung fibroblasts through the TLR4 signaling pathway, which involves downregulation of the tension protein homolog expression and activation of PI3K-Akt (He et al., 2012). Some bacteria can also cause PF by secreting cytotoxic compounds, such as *Streptococcus* pyogenes producing streptolysin (a pore-forming toxin) that triggers progressive fibrosis in a TLR4-dependent manner (Knippenberg et al., 2015), *Staphylococcus nepalensis* releasing corisin, a conserved peptide found in several Staphylococcus species, which induces apoptosis of lung epithelial cells (D'Alessandro-Gabazza et al., 2020).

Thorley and colleagues conducted a study on the impact of LPS on primary human lung macrophages and AT2 cells, which revealed distinct activation patterns for cytokine and chemokine release between the two cell types upon LPS exposure. The research demonstrated that activated alveolar epithelial cells were pivotal in generating chemokines to promote the migration of leukocytes to the peripheral lungs, and the early release of TNF-α and IL-1β by activated macrophages might contribute to the activation of alveolar epithelial cells and the production of chemokines (Thorley et al., 2007). Exposure of macrophages to the outer membrane vesicles of gram-negative bacteria triggers TLR2/4 signaling, leading to the release of IL-17, which subsequently induces the production of chemokines and growth factors in alveolar epithelial cells. This process ultimately leads to the development of PF (Yang et al., 2019) (**Table 3**).

**Table 3:** The regulation of pulmonary microbial components and secretions related to the mechanism of pulmonary fibrosis.

substance	Changing trends	mechanism	result	refer
Lipopolysaccharides (LPS)	increase	Increased collagen deposition	Interstitial thickening and decreased surface density of alveolar septa lead to fibrosis	(He et al., 2009)
	increase	Fibroblast activation, TLR4, type I procollagen α- Overexpression of SMA and p-AKT	Increased collagen deposition and pulmonary fibrosis	(de Souza Xavier Costa et al., 2017)
	increase	Downregulation of TEN expression and activation of TLR4 signaling mechanism by the (PI3K) - Akt pathway	Pulmonary fibroblast proliferation leads to pulmonary fibrosis	(He et al., 2012)
	increase	Stimulate early release of TNF from macrophages- α And IL-1 β Helps activate alveolar epithelial cells	Increased LPS stimulates macrophage secretion of cytokines and induces activation of alveolar epithelial cells, leading to pulmonary fibrosis	(Thorley et al., 2007)
streptolysin	increase	Activated TLR4 signaling mechanism	Activated TLR4 signaling mechanism promotes pulmonary fibrosis	(Knippenberg et al., 2015)
Corisin released by Staphylococcus	increase	Apoptosis of pulmonary epithelial cells	Apoptosis of pulmonary epithelial cells leading to pulmonary fibrosis	(D'Alessandro-Gabazza et al., 2020)
TLR2/4	increase	Macrophages exposed to outer membrane vesicles of Gram negative bacteria release IL-17B, inducing secretion of chemokines and growth factors by alveolar epithelial cells	Increased TLR2/4 stimulates macrophage secretion of cytokines and induces activation of alveolar epithelial cells, leading to pulmonary fibrosis	(Yang et al., 2019)

According to the studies above, LPS, which is found on the membranes of lung bacteria, activates fibroblasts and deposits collagen through TLR4 signaling. Additionally, some bacterial secretions cause apoptosis of lung epithelial cells through TLR4 signaling, which results in PF. LPS and bacterial outer membrane vesicles can promote the development of PF by releasing cytokines through immune cells such as macrophages to produce an immune response.

#### 3.2.2 Mechanism regulation of intestinal microbial metabolites and pulmonary fibrosis

A variety of intestinal microbial metabolites, including amino acids, SCFAs and bile acids, can affect fibroblasts, myofibroblasts, ECM accumulation, immune regulation and other pathways to cause PF (Wu et al., 2022).

Amino acids are prevalent metabolites in the intestines, including substances like arginine and glutamine (Gln). (I) Arginine, in particular, has been documented as having a role in collagen deposition, apoptosis, and ammonia elimination in individuals with IPF (Wang et al., 2021b). Argininosuccinate synthase 1 (ASS1) is a pivotal enzyme that governs the rate at which arginine is synthesized. Lack of ASS1 in fibroblasts from people with IPF shows that preventing these cells from getting extra arginine can slow down fibroblast growth, migration, and invasion, protecting mice from lung fibrosis caused by BLM (Li et al., 2021). High levels of arginine metabolites, including creatine, putrescine, agmatine, 4-hydroxyproline, and proline-hydroxyproline dipeptide, have been detected in IPF patients (Roque and Romero, 2021). The cationic amino acid transporter (CAT2) has been recognized as the primary arginine transporter in the majority of cells and tissues. Evaluation of CAT2-deficient mice subjected to BLM-induced fibrosis indicated that while inflammation was not reliant on CAT2 expression, the development of fibrosis indeed depended on Cat2. A deeper investigation into the mechanism unveiled that arginase activity in macrophages was partially contingent on CAT2 (Niese et al., 2010). (II) Gln, which is more abundant in the body, TGF-β1-induced differentiation and activation of myofibroblasts and production of collagen requires Gln catabolism (Bernard et al., 2018; Hamanaka et al., 2019). IPF patients show enhanced Gln catabolism in their lungs (Zhao et al., 2017). Cellular experiments show that Gln contributes to apoptosis resistance in lung fibroblasts from IPF patients. Diminished Gln metabolism has the effect of making IPF fibroblasts more susceptible to FasL-induced apoptosis, suppressing the expression of anti-apoptotic genes, and inducing changes in the epigenetic profile of these cells (Bai et al., 2019). Moreover, TGF-β1 enhances glutaminase 1 (GLS1) expression in myofibroblasts, which increases the breakdown of Gln through the SMAD3 and p38 MAPK-dependent signaling pathways (Bernard et al., 2018). These data indicate that the breakdown of Gln is a key component of the metabolic reprogramming of myofibroblasts that regulates their differentiation.

Amino acids and SCFAs have been reported to modulate the function of ILC. ILC can amplify significantly into ILC1, ILC2, and ILC3 upon contact with pathogens. The main group of ILC acting in the lungs is ILC2, which is an important mediator of type 2 immunity and is involved in the pathogenesis of PF. Elevated ILC2 promotes collagen deposition, ECM remodeling, and fibroblast activation, whereas increased SCFAs and amino acid deficiencies inhibit this process. Free fatty acid receptor 2 (FFAR2) is a G protein-coupled receptor (GPCR) receptor for SCFAs, SCFAs can suppress ILC2 proliferation through a FFAR2-independent mechanism (Sepahi et al., 2021). In contrast, intracellular deletion of the amino acid transporter proteins Slc7a5 and Slc7a8 impairs ILC2 expansion and cytokine production (Hodge et al., 2023), and the amino acid deletion affected the oxidative phosphorylation of ILC2, inhibiting the proliferation of ILC2 (Surace et al., 2021). In the lungs, blood-derived human ILC2 is able to induce upregulation of type VI collagen expression in the lungs by increasing local eosinophils and neutrophils and ILC2-derived IL-4 and IL-13, which may drive ECM remodeling and influence lung tissue destruction in CF patients (Schulz-Kuhnt et al., 2020). It was found that mice could be protected from fibrosis of the lungs by reducing TGF-β and inhibiting IL-5 and IL-13 production by ILC2 through inhibition of neurofelicitin-1 (Nrp1), a tissue-specific marker of lung ILC2 (Zhang et al., 2022). BM-derived ILC2 promotes lung fibrosis by contributing to fibroblast activation and type I collagen expression via the IL-33/ST2 pathway and TGF-β expression (Zhao et al., 2018). ILC2 also helps Th2 activation by directly producing the Th2 cytokines IL-4, IL-5, and IL-13, which promote lung inflammation and tissue fibrosis (Mikami et al., 2018).

SCFAs derive from the metabolism of dietary fiber by the intestinal microbiota, and after they are released into the intestinal lumen, they form a local immune response in the intestinal tract, supplying energy to the colonocytes, and immediately afterwards, SCFAs that remain unutilized by the gastrointestinal tract are transported through the portal vein to the liver for metabolic processing. SCFAs that are not metabolized in the liver enter the peripheral circulation and bone marrow, where they influence the development of immune cells (Wypych et al., 2019). As described in 1.1.4 of this article, dysregulation of immune cells such as macrophages, T-lymphocytes and neutrophils can cause PF, while SCFAs modulates the local and distal organ (including lung) functions of macrophages, neutrophils, ILCs and T-cells. For example, supplementation with SCFAs butyrate can restore antibiotic-induced over-responsiveness of intestinal macrophages to bacterial stimuli, producing excess inflammatory cytokines, T-cell dysfunction and persistent dysbiosis (Scott et al., 2018). Another study found that oral administration of SCFAs-acetate reduced viral load and lung inflammation in RSV-infected mice by increasing the expression of interferon-stimulated genes in the lungs to mediate the interferon-β (IFN-β) response (Antunes et al., 2019). Further ex *vivo* treatment of the patient’s respiratory cells with acetate was found to reduce the severity of RSV infection and RSV viral load by increasing viral recognition receptors and thereby modulating RIG-I expression (Antunes et al., 2022). SCFAs modulates the size and function of the mouse colonic regulatory T cell (Treg) pool and prevents colitis in GPCR receptor FFAR2-dependent manner with SCFAs (Smith et al., 2013). Increased ILC2 is known to promote collagen deposition, ECM remodeling, fibroblast activation and other processes that promote lung fibrosis, whereas it has been shown that SCFAs can inhibit ILC2 proliferation and inhibit lung fibrosis through FFAR2-independent mechanism (Sepahi et al., 2021).

The inhalation of minimal quantities of bile acids induced EMT in alveolar epithelial cells, the mesenchymal markers α-smooth muscle actin (α-SMA) and waveform protein were upregulated, and the epithelial markers E-calmodulin and cytokeratin were downregulated. The expression of fibrosis mediators, including TGF-β1, CTGF, VEGF, basic fibroblast growth factor (bFGF) and thrombospondin is significantly elevated in the lungs of rats exposed to trace amounts of bile acids (Chen et al., 2017). Inhaling small quantities of bile acids triggers the activation of alveolar epithelial cells and myofibroblasts by means of the bile acid receptor FXR, thus activating the TGF-β1/SMAD3 signaling pathway (Chen et al., 2016) (**Table 4**).

**Table 4:** Mechanism regulation of intestinal microbial metabolites and pulmonary fibrosis.

substance	Changing trends	mechanism	result	refer
arginine	increase	Cell apoptosis and ammonia removal, collagen deposition	Collagen deposition and pulmonary fibrosis formation	(Wang et al., 2021b)
	increase	Depletion of arginine weakens the proliferation, migration, and invasion of fibroblasts, thereby slowing down pulmonary fibrosis	Fibroblast activation, causing pulmonary fibrosis	(Li et al., 2021)
	increase	Proline from arginine metabolism is a rate limiting substrate for collagen synthesis, which is crucial for collagen precipitation in pulmonary fibrosis	Collagen deposition, causing pulmonary fibrosis	(Niese et al., 2010; Roque and Romero, 2021)
glutamate	increase	Glutamic acid glutamine cycle participates in collagen production in myofibroblasts	Collagen production, causing pulmonary fibrosis	(Bernard et al., 2018; Hamanaka et al., 2019)
	increase	Glutamine promotes anti apoptosis of IPF fibroblasts through epigenetic regulation of apoptosis inhibitory proteins	Fibroblast apoptosis, ultimately leading to pulmonary fibrosis formation	(Bai et al., 2019)
bile acid	increase	Bile acid stimulates fibrotic mediators to activate TGF- β 1/SMAD3 signaling pathway and bile acid receptor FXR or induction of activation of alveolar epithelial cells and lung fibroblasts	Collagen production, causing pulmonary fibrosis	(Chen et al., 2016; Chen et al., 2017)
short-chain fatty acids (SCFA)	increase	inhibit ILC2 proliferation through a non-Ffar2(G protein-coupled receptor) receptor mediated mechanism,	Inhibiting various inflammatory reactions, inhibiting collagen deposition, and extracellular matrix remodeling to alleviate pulmonary fibrosis	(Sepahi et al., 2021) (Schulz-Kuhnt et al., 2020)
amino acids	decrease	intracellular deletion of the amino acid transporter proteins Slc7a5 and Slc7a8 impairs ILC2 expansion and cytokine production	Inhibiting various inflammatory reactions, inhibiting collagen deposition, and extracellular matrix remodeling to alleviate pulmonary fibrosis	(Hodge et al., 2023) (Schulz-Kuhnt et al., 2020)

From the above reports, we can see that amino acids play an important role in apoptosis, fibroblast differentiation and collagen deposition during the formation of PF, and in the immune response, amino acid deletions can inhibit the formation of PF by suppressing the function of ILC2. Similarly, SCFAs can also reduce PF by inhibiting collagen deposition and fibroblast activation through inhibiting the function of ILC2, and SCFAs can regulate a variety of immune cells through the circulatory system to reduce PF in addition to generating an immune response locally in the intestinal tract. Bile acids may induce activation of alveolar epithelial cells and myofibroblasts through activation of the TGF-β1/SMAD3 signaling pathway and high expression of various fibrous mediators.

## 4 Treatment

The main regulatory mechanisms of the microbiota, metabolites and immunomodulation in PF have been described in the above paragraphs and the aim of the study is to better diagnose and treat the disease. Many microorganisms and metabolites or immunomodulation, present a positive feedback loop on PF, where microorganisms have a good influence on the process and play a positive role in the treatment of PF. For example, researchers have used beneficial bacteria or fecal microbiome transplants to alleviate the condition of patients with PF, or added algal blue proteins to regulate the microbiota to go back to the normal state to reduce PF, or used microbial metabolites such as SCFAs and amino acids to regulate the immune cells, reduce collagen deposition, and inhibit the differentiation of fibroblasts to reduce PF, and also through certain components or metabolites of microbes to regulate the immune system to inhibit the inflammatory response to alleviate PF. This will be described below.

### 4.1 Treatment of pulmonary fibrosis by modulating the gut microbiome

Microbial alterations are closely related to PF, so treating PF by modulating gut microbes is one of the directions of research, and the administration of compounds from traditional Chinese medicine, algae, *Amygdalus mongolica* oil, and different bacteria have been tested in PF models to attenuate lung fibrosis.

The Qingwen Gupi decoction (QGT) is a Chinese herbal remedy comprising a combination of Shengjiang powder (consisting of cicada slough, stiff silkworm, turmeric, and cooked army) and Xiao Chaihu decoction (composed of Bupleurum, *Scutellaria*, Codonopsis, ginger, forsythia, raw licorice, fresh ginger, and jujube). Shengjiang powder is effective in the management of Viral Pneumonia through the regulation of the inflammatory immune response, while Xiao Chaihu decoction can impede the development of fibrosis in the pancreas and liver. It was found that QGT attenuated symptoms of BLM-induced PF in rat model by altering the intestinal microbiota and metabolic pathways to attenuate inflammatory responses, oxidative stress, fibroblast hyperproliferation, and collagen production in the ECM (Gao et al., 2023). Astragalus polysaccharide (APS) is a natural compound extracted from the traditional Chinese medicine *Astragalus membranaceus*, which has anti-inflammatory and antioxidant properties, and can reduce collagen deposition and EMT. APS was found to improve PF by increasing the proportion of probiotics and decreasing the proportion of harmful bacteria, and by modulating metabolic pathways to alleviate intestinal dysbiosis induced by PF, and to reduce the extent of lung tissue damage and collagen deposition (Wei et al., 2023). Bu-Fei-Huo-Xue capsules, containing *Astragalus mongholicus* Bunge and Paeonia *anomala subsp*. are employed in clinical practice for the treatment of PF. It has the capability to influence the proportions of gut microbiota like *Lactobacillus*, *Lachnospiraceae*_NK4A136_*group*, and *Romboutsia*, and it can decrease collagen accumulation in a mouse model of PF (Hu et al., 2023). Qi-Long-Tian (QLT) capsule is an herbal formulation that consists of San Qi, Di Long and Hong Jingtian. It was found to significantly increase the relative abundance of Bacteroidia and decrease the relative abundance of Clostridia, and intervene in PF by modulating the differential genera of intestinal flora, increasing the secretion of immunoglobulins, repairing the intestinal mucosal barrier, reducing the entry of LPS into the bloodstream, and decreasing the secretion of inflammatory factors in the serum (Zhang et al., 2023).

By X-ray irradiation and BLM-induced PF mice, it was found that the diversity of the microbiota in the lungs and intestines showed opposite trends, but the composition of the microbiota showed similar trends. Intervention with phycocyanin, a protein derived from blue-green algae, regulated the composition of lung and gut microbiota into a normal state and decreased the level of LPS, resulting in a significant decrease in the abundance of inflammation-related bacteria and an increase in the abundance of beneficial bacteria (Xie et al., 2019; Li et al., 2020). Li and others demonstrated that Amygdalus mongolica oil increased the abundance of intestinal flora, such as *Duncaniell*, *Desulfovibrio*, *Peptococcaceae_unclassified*, *Dubosiella*, *Tyzzerella*, *Lachnospiraceae _NK4A136_group*, *Lactobacillus* and *Clostridiales_unclassified*, increasing intestinal flora diversity, reducing oxidative and inflammatory damage, modulating the ECM and thus improving PF (Li et al., 2022).

*Lactobacillus reuteri* probiotics improve digestive health and reduce deformable bacterial populations in the intestinal microbiome of patients with CF (del Campo et al., 2014). The addition of *Lactobacillus reuteri* caused an increase in the diversity of the intestinal flora and a decrease in the total bacterial density, accompanied by a significant increase in Firmicutes and a decrease in the proportion of Proteobacteria, which in turn caused a significant decrease in the intestinal levels of fecal calprotectin (FCP), which can help to control the deterioration of the disease and safeguard the quality of life of patients with cystic fibrosis. As a result, levels of FCP in the intestine have significantly decreased (Van Biervliet et al., 2018). An experimenter administered BLM to induce mouse PF models in two environments: ABSL-1 (no experiments involving infectious sources; mice only have symbiotic organisms) and ABSL-2 (experiments involving infectious pathogens with moderate potential danger to personnel). It was found that the intestines of mice in ABSL-1 contain many *Lactobacillus* that can reduce immune activity, such as *Dubosiella newyorkensis*, *Staphylococcus Nepalensis*, ABSL-2 also contains *Romboutsia ilealis*, which is positively correlated with immune activation. The PF guided by ABSL-2 is more severe than that guided by ABSL-1, indicating that beneficial bacteria are beneficial in reducing PF (Chioma et al., 2022).

Fretheim and others studied SSc patients with intestinal symptoms and performed fecal microbial transplantation (FMT) with human gut microbiota (Ruminococcaceae, Lachnospiraceae and Eggerthellaceae), which are predominantly butyric acid-producing, and the mobilization of neutrophils by FMT reflected the activation of an adaptive immune response. A comparison of the gut microbiome before and after transplantation was made, and it was found that gastrointestinal symptoms and lung function were well improved after FMT, so modulation of the gut microbiota could be effective in improving lung function in this patient (Fretheim et al., 2020). Polysaccharide A (PSA), the immunomodulatory molecule of *Bacteroides fragilis*, modulates the inflammatory response and slows down the progression of PF by mediating the conversion of CD4^+^ T cells into Foxp3^+^ Tregs cells to produce IL-10 and TGF-β2 through the expression of TLR2 (Round and Mazmanian, 2010) (**Table 5**).

**Table 5:** Treatment of pulmonary fibrosis by modulating gut microbiome.

substance	The trend of change	mechanism	Result	References
Qingwen Gupi decoction	administration of medication	Change the gut microbiota and metabolic pathways	Reduce inflammation, oxidative stress, excessive proliferation of fibroblast, collagen production in extracellular matrix, and alleviate fibrosis.	(Gao et al., 2023)
Astragalus polysaccharide (APS)	administration of medication	increasing the proportion of probiotics and decreasing the proportion of harmful bacteria	attenuating the degree of damage and collagen deposition in lung tissue, and alleviate fibrosis.	(Wei et al., 2023)
Bu-Fei-Huo-Xue capsule (BFHX)	administration of medication	affect the relative abundance of intestinal microbiota such as Lactobacillus, Lachnospiraceae_NK4A136_group, and Romboutsia.	Reduced collagen deposition in mice with pulmonary fibrosis model	(Hu et al., 2023)
Qi-Long-Tian (QLT)	administration of medication	increase the relative abundance of inflammation-inhibiting anaphylactic bacilli and decrease the relative abundance of inflammation-promoting clostridia	ntervening in pulmonary fibrosis by modulating the differential genera of intestinal flora and modulating the immune response	(Zhang et al., 2023)
phycocyanin	administration of medication	Intervene to regulate the composition of the lung and intestinal microbiota, bringing them back to a normal state	Reduce the level of triglycerides, resulting in a significant decrease in abundance of inflammation-related bacteria and an increase in abundance of beneficial bacteria and alleviate pulmonary fibrosis.	(Xie et al., 2019; Li et al., 2020)
Amygdalus mongolica oil	administration of medication	increased the abundance of intestinal flora, such as Duncaniell, Desulfovibrio, Peptococcaceaeunclassified, Dubosiella, Tyzzerella, Lachnospiraceae NK4A136 group, Lactobacillus, Clostridiales unclassified, etc.	increasing intestinal flora diversity, reducing oxidative and inflammatory damage, modulating the extracellular matrix.	(Li et al., 2022).
Lactobacillus reuteri probiotic preparation	administration of medication	The diversity of gut microbiota increased while the overall bacterial density decreased, accompanied by a significant increase in the population of Firmicutes and a decrease in the proportion of Proteobacteria.	Causing a significant decrease in fecal calprotectin levels, improving digestive system health, and controlling the progression of cystic fibrosis patient's condition.	(del Campo et al., 2014; Van Biervliet et al., 2018)
Fecal microbiota transplantation	transplant	Lung function has been effectively improved.	Fecal microbiota transplantation can improve lung function in patients with SSc.	(Fretheim et al., 2020)
Polysaccharide A (PSA) produced by human symbiotic bacteria, bacteroides fragilis	administration of medication	Through the expression of Toll-like receptor 2 (TLR2), it mediates the transformation of CD4+ T cells into Foxp3+ Tregs cells, producing IL-10 and TGF-β2.	Regulate inflammation response and slow down the progression of pulmonary fibrosis.	(Round and Mazmanian, 2010)

The article mentioned earlier says that traditional Chinese medicine, algae, and Amygdalus mongolica oil are mostly used to treat PF by reducing the growth of too many fibroblasts and the buildup of ECM. This is achieved through the promotion of beneficial bacterial populations and the reduction of harmful bacteria, the enhancement of intestinal flora diversity, heightened immunoglobulin levels, and the alleviation of oxidative stress and inflammatory damage. Beneficial bacteria can also be added directly to attenuate PF by modulating the immune cells, such as through T-cells and neutrophils. Immunomodulation is an important part of the gut-lung axis and is inextricably linked to microbial regulatory processes. However, there are fewer studies related to the treatment of PF through the immune system, which is a direction for future research.

### 4.2 Treatment of pulmonary fibrosis by metabolites

It is known that microbial metabolites have an important role in the mechanism of PF formation, and a review of articles has revealed that common microbial metabolites SCFAs and certain amino acid metabolites are helpful in the treatment of PF. SCFAs are the more common microbial metabolites that have a role in modulating immune cell function in the context of PF, and the hypoxic environment of the CF airways permits the persistence of anaerobic bacteria that can produce SCFAs by fermentation. It has been shown that SCFAs causes excessive IL-8 production in CF airways colonized with anaerobic bacteria by upregulating the short-chain fatty acid receptor GPR41 (Mirković et al., 2015), that SCFAs contributes to CF-specific alterations in airway infections, inflammatory responses and bacterial growth, and that high concentrations of SCFAs reduce the proliferation of an alveolar epithelial cell line (A549) (Ghorbani et al., 2015). Butyrate is a bacterial metabolite and a type of SCFAs. Research has found that butyrate affects the composition of the fecal microbiota (phyla Actinobacteria and Bacteroidetes, genera *Bifidobacterium* and *Ruminococcus_g2*), inhibits the response of TGF-β1, regulates macrophage differentiation, and inhibits the indirect and direct anti-fibrosis performance of histone deacetylase 3 in fibroblasts (Park et al., 2021). Similarly, studies have claimed that butyrate prevents TGF-β1-induced changes in mitochondrial dynamics and energy metabolism induced during the differentiation of pulmonary myofibroblasts (Lee et al., 2021). Valproic acid is also a type of SCFAs. Noguchi and others found *in vitro* that Sodium Valproate can reduce the acetylation of histone H3K27 and partially inhibit the EMT induced by TGF-β, thus directly reducing lung fibrosis (Noguchi et al., 2015). Valproate and butyrate can attenuate PF by blocking threonine kinase (Akt) expression, leading to apoptosis and TGF-β1 production (Chen et al., 2006; Sakai and Tager, 2013).

There is a wide range of amino acids that are known to affect PF, and therapeutically, the amino acid metabolite 5-methoxytryptophan (5-MTP), combinations of L-arginine and L-norvaline, and tryptophan derivatives have been reported in the treatment of PF. The use of 5-MTP, a tryptophan metabolite, has yielded encouraging outcomes in mouse models for treating PF. The administration of 5-MTP has been shown to enhance lung functionality and alveolar structure by diminishing the buildup of myofibroblasts and the deposition of ECM. This is achieved by inhibiting fibroblast differentiation into myofibroblasts and reducing ECM protein expression. In addition, stimulation of human lung fibroblasts with TGF-β1 has been shown to decrease fibroblast proliferation and migration, and to suppress PF by downregulating the phosphorylation of TGF-β/SMAD3 and PI3K/AKT signaling pathways (Fang et al., 2020). The combination of L-arginine and L-norvaline reversed weight loss, decreased lung index and hydroxyproline, ameliorated BLM-induced histopathological damage in the lungs, and dynamically corrected abnormal changes in Tregs, Th17, γδT and Tregs/Th17. These results suggest that the combination of L-arginine and L-norvaline can inhibit BLM-induced PF progression through the modulation of its immune system (Gao et al., 2019). Activation of aryl hydrocarbon receptor (AhR) signaling by various ligands (such as tryptophan derivatives) induces hyperimmune responses and participates in autoimmunity, and in a BLM-induced fibrosis model, stimulation of AhR signaling attenuates PF by increasing Tregs and suppressing inflammatory T cell subsets (Takei et al., 2020) (**Table 6**).

**Table 6:** Treatment of pulmonary fibrosis by metabolites.

substance	The trend of change	mechanism	Result	References
SCFA	administration of medication and add	SCFA leads to IL-8 overproduction in anaerobically colonised CF airways through upregulation of the short-chain fatty acid receptor GPR41, reduce the proliferation of an alveolar epithelial cell line (A549)	Directly reduce pulmonary fibrosis.	(Mirković et al., 2015) (Ghorbani et al., 2015)
Butyrate	add	prevents TGF-β1-induced changes in mitochondrial dynamics and energy metabolism induced during differentiation of pulmonary myofibroblasts	Directly reduce pulmonary fibrosis.	(Lee et al., 2021)
	administration of medication	This means that the composition of the microbial population in feces, specifically the phyla Actinobacteria and Bacteroidetes, genera Bifidobacterium and Ruminococcus_g2, is affected by this factor. Additionally, this factor is able to suppress the responsiveness of transforming growth factor β1, regulate the differentiation of macrophages, and inhibit histone deacetylase 3.	"Influence on fibroblast, showing indirect and direct antifibrotic effects."	(Park et al., 2021)
Sodium valproate	add	By improving the reduction of histone H3K27 acetylation, partially inhibit TGF-β-induced epithelial mesenchymal transition.	Directly reduce pulmonary fibrosis.	(Noguchi et al., 2015)
histone deacetylase inhibitors such as Valproic acid and butyrate	add	By inhibiting Akt/Protein kinase B gene, the nuclear factor kappa B (NF-κB) entry into the nucleus is reduced and the production expression of TGF-β1 is decreased.	The combination of butyric acid and propionic acid reduces the number of NF-κB entering the nucleus and decreases the expression of TGF-β1 to alleviate pulmonary fibrosis.	(Chen et al., 2006; Sakai and Tager, 2013)
5-Methoxytryptophan (5-MTP)	administration of medication and add	By down-regulating the phosphorylation of the TGF-β/SMAD3 and PI3K/AKT signaling pathways, inhibiting the differentiation, proliferation, and migration of fibroblasts into myofibroblasts, and suppressing the protein expression of ECM in vivo and in vitro.	Significantly reducing the accumulation of myofibroblasts and ECM deposition, improving lung function and reducing destruction of alveolar structure, reducing pulmonary fibrosis.	(Fang et al., 2020).
The combination of L-Arginine and L-Norvaline	administration of medication	Correcting the abnormal changes of Tregs, Th17, γδT and Tregs/Th17 can dynamically reverse weight loss, reduce lung index and hydroxyproline, and improve BLM-induced pulmonary histological damage.	The combination of L-arginine and L-carnitine has an inhibitory effect on the progression of BLM-induced pulmonary fibrosis, possibly due to their corrective effects on immune imbalance, arginine metabolism disorders, and abnormal cross-talk in pulmonary fibrosis progression.	(Gao et al., 2019)
AhR ligand such as tryptophan derivatives	injected intraperitoneally	Activation of aryl hydrocarbon receptor (AhR) signaling increases Tregs and suppresses inflammatory T cell subsets.	Tryptophan derivatives induce hyperimmune response through AhR signaling and participate in autophagy to alleviate pulmonary fibrosis.	(Takei et al., 2020)

SCFAs, including butyrate and valproate, can inhibit fibroblast differentiation and apoptosis by inhibiting TGF-β1, improving histone H3K27 acetylation reduction, hindering Akt expression, regulating macrophage differentiation, and inhibiting histone deacetylase 3, resulting in a therapeutic effect on PF. They also attenuate lung fibrosis by reducing the infectious and inflammatory response through immune modulation. Certain amino acid metabolites also inhibit fibroblast differentiation and ECM deposition, and regulating immunity can treat PF by increasing Tregs and suppressing inflammatory T-cell subsets.

## 5 Outlook

PF is a highly fatal and progressive ILD caused by multiple environmental factors that can present symptoms in the pathological process of multiple respiratory system diseases. The pathogenesis of PF has many mechanisms, including alveolar epithelial cell inefficacy, endothelial cell disorders, fibroblast heterogeneity and plasticity, immune cell regulation, integrin-mediated activation of the TGF-β pathway, cytokine regulation, and, in recent years, microbial ecological regulation of the gut-lung axis, which has been found to be a pathogenetic mechanism and a modifying factor of PF disease. So far, it has been shown that the gut-lung axis is closely linked to the development of PF. It has also been reported that the gut microbiome, metabolites, and immune pathways connected to them can be used to treat PF. Research on the mechanisms of the gut-lung axis and PF is still at an early stage of exploration. Microbiological studies have focused on the changes in the lung and gut microbiota in PF, which are reflected in the decrease in microbial diversity and the increase in microbial loads. However, there is a lack of studies on the mechanisms by which the microbial alterations mentioned above specifically affect the immune responses leading to PF, and there is a lack of direct studies on the interaction between the gut microorganisms and lung microorganisms in the development of PF, which is one of the future directions of the research. Microbial metabolites in regulating the immune system, mainly T cells, macrophages neutrophils, etc., however, more relevant immune cells and immune pathways are less studied and are a focus for future research. Therapeutically, there are mainly studies related to traditional Chinese medicine, herbal extracts, algae, Amygdalus mongolica oil, different beneficial bacteria, SCFAs, and certain amino acid metabolites, but there are still fewer studies on the specific mechanisms and pathways through which the various drugs work. Although there are some shortcomings in the study of the gut-lung axis mechanism related to PF, it also provides ideas for subsequent research on the mechanism and treatment. In the future research of PF, in addition to the microorganisms, metabolites and immune functions related to the gut-lung axis, the specific mechanisms should be studied in depth, starting from the specific mechanisms, to provide a theoretical and research basis for finding more effective diagnostic and therapeutic means.

## Author contributions

YD: Writing – original draft. LH: Writing – original draft. ZZ: Writing – review & editing. FY: Writing – review & editing. QM: Writing – review & editing. YZ: Writing – review & editing. XZ: Writing – review & editing. XL: Writing – review & editing.
